# Supplementary material for: Age-dependent formation of TMEM106B amyloid filaments in human brains
Source: Nature. 2022 Mar 28;605(7909):310–4. doi: 10.1038/s41586-022-04650-z (PMC9095482; doi:10.1038/s41586-022-04650-z)
Supplement: Supplementary file 1 — This file contains source images for western blots shown in Fig. 2. [file 41586_2022_4650_MOESM1_ESM.pdf]

nature portfolio

https://doi.org/10.1038/s41586-022-04650-z

Supplementary information

Age-dependent formation of TMEM106B amyloid filaments in human brains

In the format provided by the authors and unedited

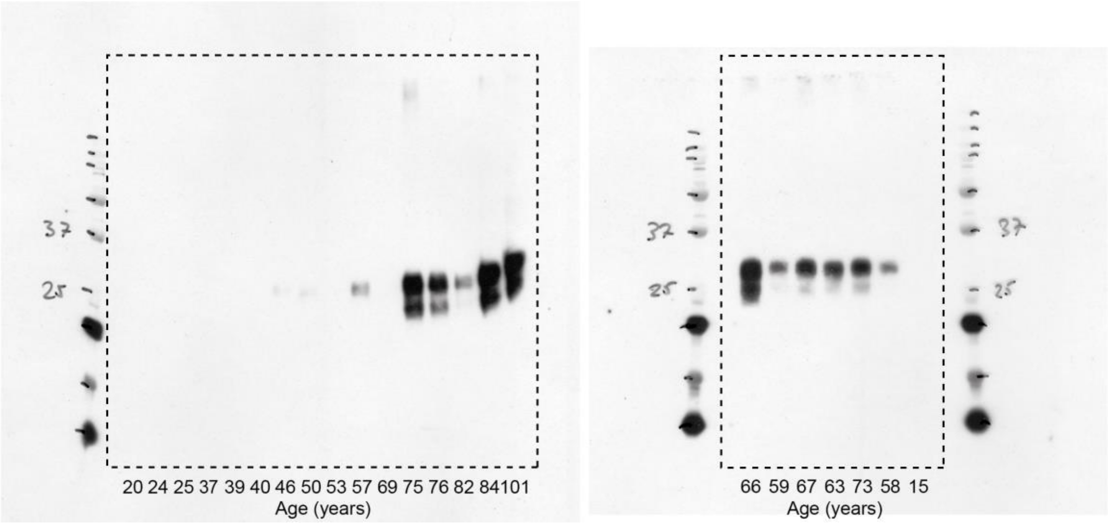

Supplementary Figure 1. Source images for Western blots shown in Figure 2.
