# Supplementary material for: Age-dependent formation of TMEM106B amyloid filaments in human brains
Source: Nature. 2022 Mar 28;605(7909):310–4. doi: 10.1038/s41586-022-04650-z (PMC9095482; doi:10.1038/s41586-022-04650-z)
Supplement: Supplementary file 2 — Reporting Summary [file 41586_2022_4650_MOESM2_ESM.pdf]

nature research

Corresponding author(s): Michel Goedert, Sjors H.W. Scheres

Last updated by author(s): Mar 2, 2022

Reporting Summary

Nature Research wishes to improve the reproducibility of the work that we publish. This form provides structure for consistency and transparency in reporting. For further information on Nature Research policies, see Authors & Referees and the Editorial Policy Checklist.

Statistics

For all statistical analyses, confirm that the following items are present in the figure legend, table legend, main text, or Methods section.

n/a	Confirmed	
☐	☒	The exact sample size (*n*) for each experimental group/condition, given as a discrete number and unit of measurement
☐	☒	A statement on whether measurements were taken from distinct samples or whether the same sample was measured repeatedly
☒	☐	The statistical test(s) used AND whether they are one- or two-sided<br>*Only common tests should be described solely by name; describe more complex techniques in the Methods section.*
☒	☐	A description of all covariates tested
☒	☐	A description of any assumptions or corrections, such as tests of normality and adjustment for multiple comparisons
☒	☐	A full description of the statistical parameters including central tendency (e.g. means) or other basic estimates (e.g. regression coefficient) AND variation (e.g. standard deviation) or associated estimates of uncertainty (e.g. confidence intervals)
☒	☐	For null hypothesis testing, the test statistic (e.g. *F*, *t*, *r*) with confidence intervals, effect sizes, degrees of freedom and *P* value noted<br>*Give P values as exact values whenever suitable.*
☒	☐	For Bayesian analysis, information on the choice of priors and Markov chain Monte Carlo settings
☒	☐	For hierarchical and complex designs, identification of the appropriate level for tests and full reporting of outcomes
☒	☐	Estimates of effect sizes (e.g. Cohen's *d*, Pearson's *r*), indicating how they were calculated

Our web collection on statistics for biologists contains articles on many of the points above.

Software and code

Policy information about availability of computer code

Data collection	EPU (v1.11.1 and v2.3.079)
Data analysis	RELION (v3.1 and v4.0), CTFFIND (v4.1), COOT (v0.9), Chimera (v1.8.1), ChimeraX (v1.2), ISOLDE (v1.2), Adobe Photoshop 22.1.1, ImageJ (v2.1.0/1.53c)

For manuscripts utilizing custom algorithms or software that are central to the research but not yet described in published literature, software must be made available to editors/reviewers. We strongly encourage code deposition in a community repository (e.g. GitHub). See the Nature Research guidelines for submitting code & software for further information.

Data

Policy information about availability of data

All manuscripts must include a data availability statement. This statement should provide the following information, where applicable:

- Accession codes, unique identifiers, or web links for publicly available datasets
- A list of figures that have associated raw data
- A description of any restrictions on data availability

Raw cryo-EM micrographs are available in the Elecron Microscopy Public Image Archive (EMPIAR), entry numbers EMPIAR-10916 for case 1 (sporadic AD), EMPIAR-10968 for case 18 (MSA), EMPIAR-10358 for case 19 (MSA) and EMPIAR-10357 for case 17 (MSA). Cryo-EM maps have been deposited in the Electron Microscopy Data Bank (EMDB) under accession numbers EMD-14174 for I-s filaments of case 1, EMD-14176 for I-d filaments of case 18, EMD-14187 and EMD-14188 for IIa-s and IIb-s of case 19, respectively, and EMD-14189 for III-s of case 17. Refined atomic models have been deposited in the Protein Data Bank (PDB) under accession numbers 7QVC for I-s of case 1, 7QVF for I-d of case 18, 7QWG and 7QWL for IIa-s and IIb-s of case 19, respectively, and 7QWM for III-s of case 17.

Field-specific reporting

Please select the one below that is the best fit for your research. If you are not sure, read the appropriate sections before making your selection.

☒ Life sciences ☐ Behavioural & social sciences ☐ Ecological, evolutionary & environmental sciences

For a reference copy of the document with all sections, see nature.com/documents/nr-reporting-summary-flat.pdf

Life sciences study design

All studies must disclose on these points even when the disclosure is negative.

Sample size	Frontal cortex from case 1 (sporadic AD), case 2 (inherited AD), case 3 (early-onset AD), case 4 (pathological ageing), cases 5 and 6 (both CBD), case 10 (LNT), cases 15 and 16 (both DLB), case 20 (FTLD-TDP-A), case 21 (FTLD-TDP-43), cases 23-25 and 12 additional cases (all neurologically normal individuals), temporal cortex from case 5 (FTDP-17T), case 14 (inherited PD), nucleus accumbens from cases 8 and 9 (both AGD), hippocampus from case 11 (ARTAG), cingulate cortex from case 12 (PD), amygdala from case 13 (PDD), putamen from cases 17, 18 and 19 (all MSA), and motor cortex from case 22 (ALS). All these samples were chosen based on availability of tissue (maximum available sample size).
Data exclusions	Pre-established common image classification procedures (S.H.W. Scheres, J. Struc. Biol. 180: 519-530, (2012)) were employed to select particle images with the highest resolution content in the cryo-EM reconstruction process. Details of the number of selected images are given in Extended Data Table 2.
Replication	All attempts at replication were successful. At least three independent biological repeats per experiment where representative data is shown.
Randomization	Randomisation was not performed, as it would not reduce any bias in this study, where samples are limited by brain availability. Therefore, samples were allocated in one experimental group (frontal cortex from cases 1-6, 10, 15, 16, 20, 21, 23-25, temporal cortex from cases 5 and 14, nucleus accumbens from cases 8 and 9, hippocampus from case 11, cingulate cortex from case 12, amygdala from case 13, putamen from cases 17-19, and motor cortex from case 22) based on neuropathological examination.
Blinding	Blinding was not performed, as the perceived risk of detection/performance bias was deemed negligible.

Reporting for specific materials, systems and methods

We require information from authors about some types of materials, experimental systems and methods used in many studies. Here, indicate whether each material, system or method listed is relevant to your study. If you are not sure if a list item applies to your research, read the appropriate section before selecting a response.

Materials & experimental systems

n/a	Involved in the study	
☐	☒	Antibodies
☒	☐	Eukaryotic cell lines
☒	☐	Palaeontology
☒	☐	Animals and other organisms
☐	☒	Human research participants
☒	☐	Clinical data

Methods

n/a	Involved in the study	
☒	☐	ChIP-seq
☒	☐	Flow cytometry
☒	☐	MRI-based neuroimaging

Antibodies

Antibodies used	Primary antibody used for Western blotting was rabbit polyclonal TMEM239 used at 1:2,000. Primary antibodies used for immunohistochemistry were rabbit polyclonal TMEM239 used at 1:500 and rabbit polyclonal A303-439A (Bethyl Laboratories, catalogue number: A303-439A) used at 1:250.
Validation	TMEM239 validated against human TMEM106B residues 239-250 in Extended Data Figure 7. A303-439A was validated against recombinant human TMEM106B (Satoh, Ji. et al. Alz. Res. Therapy 6, 17 (2014).

Human research participants

Policy information about studies involving human research participants

Population characteristics	See Table 1, Methods section, Extended Data Table 1, and Supplementary Table 1. Age at death: 79, 67, 58, 59, 74, 79, 55, 85, 90, 66, 85, 87, 64, 67, 74, 73, 85, 70, 68, 66, 65, 63, 75, 84, 101, 15, 20, 24, 25, 37, 39, 40, 46, 50, 57, 69, 76, 82; Gender: 16x female, 22x male; Diagnoses: 3x AD (sporadic, inherited, and early-onset), 1x PA, 2x CBD, 1x FTDP-17T, 2x AGD, 1x LNT, 1x

	ARTAG, 2x PD (sporadic and inherited), 1x PDD, 3x DLB (2x sporadic and early-onset), 3x MSA, 1x FTLD-TDP-A, 1x FTLD-TDP-C, 1x ALS, 15x neurologically normal individuals.
Recruitment	Samples were selected based on neuropathological examination and brain tissue availability, which is unlikely to have impacted the results.
Ethics oversight	The studies carried out at Indiana University, Tokyo Metropolitan Institute of Medical Science, UCL Queen Square Institute of Neurology, Edinburgh Brain and Tissue Bank, Toronto University, Vienna Medical University, Rotterdam University and Keio University were approved through the ethical review processes at each university's Institutional Review Board (IRB). Informed consent was obtained from the patients' next of kin.

Note that full information on the approval of the study protocol must also be provided in the manuscript.
